# LAIR-1 and PECAM-1 function via the same signaling pathway to inhibit GPVI-mediated platelet activation

**DOI:** 10.1016/j.rpth.2024.102557

**Published:** 2024-08-23

**Authors:** Christopher W. Smith, Zoltan Nagy, Mitchell J. Geer, Jeremy A. Pike, Pushpa Patel, Yotis A. Senis, Alexandra Mazharian

**Affiliations:** 1 Institute of Cardiovascular Sciences, College of Medical and Dental Sciences, University of Birmingham, Birmingham, United Kingdom; 2 Institute of Experimental Biomedicine, University Hospital and Rudolf Virchow Center, University of Würzburg, Würzburg, Germany; 3 Laura and Isaac Perlmutter Cancer Center, New York University School of Medicine, NYU Langone Health, New York, New York, USA; 4 Institut National de la Santé et de la Recherche Médicale (INSERM), Etablissement Français du Sang (EFS) Grand-Est, Unité Mixte de Recherche (UMR)-S 1255, Université de Strasbourg, Strasbourg, France

**Keywords:** hyperactive platelets, ITIM-containing receptors, LAIR-1, PECAM-1, thrombosis

## Abstract

**Background:**

Inhibition of platelet responsiveness is important for controlling thrombosis. It is well established that platelet endothelial cell adhesion molecule-1 (PECAM-1) serves as a physiological negative regulator of platelet-collagen interactions. We recently demonstrated that leukocyte-associated immunoglobulin-like receptor-1 (LAIR-1) is a negative regulator of platelet production and reactivity. It is however not known if LAIR-1 and PECAM-1 function in the same or different inhibitory pathways.

**Objectives:**

In this study, we investigated the role of LAIR-1 alongside PECAM-1 in megakaryocyte development and platelet production and determined the functional redundancy through characterization of a LAIR-1/PECAM-1 double knockout (DKO) mouse model.

**Methods:**

LAIR-1 and PECAM-1 expression in megakaryocytes were evaluated by western blotting. Megakaryocyte ploidy and proplatelet formation were evaluated by flow cytometry and fluorescent microscopy. Platelet function and signalling were compared in wild-type, *LAIR-1^^−/−^^*, *PECAM-1^^−/−^^* and DKO mice using aggregometry, flow cytometry and western blotting. Thrombosis was evaluated using the FeCl__3__ carotid artery model.

**Results:**

We show that LAIR-1/PECAM-1 DKO mice exhibit a 17% increase in platelet count. Bone marrow-derived megakaryocytes from all 3 mouse models had normal ploidy *in vitro*, suggesting that neither LAIR-1 nor PECAM-1 regulates megakaryocyte development. Furthermore, relative to wild-type platelets, platelets derived from LAIR-1, PECAM-1, and DKO mice were equally hyperresponsive to collagen *in vitro*, indicating that LAIR-1 and PECAM-1 participate in the same inhibitory pathway. Interestingly, DKO mice exhibited normal thrombus formation *in vivo* due to DKO mouse platelets lacking the enhanced Src family kinase activation previously shown in platelets from LAIR-1-deficient mice.

**Conclusion:**

Findings from this study reveal that LAIR-1 and PECAM-1 act to inhibit GPVI-mediated platelet activation via the same signaling pathway. Mice lacking LAIR-1 and PECAM-1 do not however exhibit an increase in thrombus formation despite minor increase in platelet count and reactivity to collagen. This study adds to the growing evidence that immunoreceptor tyrosine-based inhibition motif–containing receptors are important regulators of platelet count and function.

## Essentials

• Mice lacking immunoreceptor tyrosine-based inhibition motif–containing receptors LAIR-1 and PECAM-1 have a marginal increase in platelet count.
• Platelets from LAIR-1/PECAM-1 double knockout mice hyperrespond to collagen *in vitro.*
• Thrombus formation and platelet Src family kinase activity appear to be rescued in double knockout mice.
• LAIR-1 and PECAM-1 inhibit platelet activation via the same signaling pathway.

## 1 Introduction

Rapid platelet activation and thrombus formation are essential to prevent blood loss at sites of vascular injury but must be tightly controlled to prevent pathological thrombus formation. Following initial tethering by the GPIb-IX-V von Willebrand factor receptor complex, strong platelet activation is mediated by the immunoreceptor tyrosine-based activation motif (ITAM)-containing receptor complex GPVI-Fc receptor γ-chain (FcRγ) binding to exposed subendothelial collagen. Collagen-mediated GPVI-FcRγ clustering induces Src family kinase (SFK)-mediated phosphorylation of the conserved tyrosine residues within the FcR γ-chain ITAM [1]. This supports the recruitment and activation of Syk tyrosine kinase and assembly of the linker for activation of T cells signalosome, which, through PLCγ2 and protein kinase C activation and intracellular calcium mobilization, enables platelet aggregation through platelet integrin activation, granule secretion, and shape change [2,3].

Platelet endothelial cell adhesion molecule-1 (PECAM-1) is an immunoreceptor tyrosine-based inhibition motif (ITIM)-containing receptor that inhibits collagen-induced GPVI-FcRγ signaling through a well-established mechanism [[4], [5], [6]]. Highly expressed in platelets and endothelial cells, PECAM-1 undergoes *trans-*homophilic interactions, resulting in tyrosine phosphorylation and recruitment of the nontransmembrane Src homology 2 domain-containing tyrosine phosphatases Shp1 and Shp2 to ITIMs and immunoreceptor tyrosine-based switch motifs [[7], [8], [9]]. Mice deficient in PECAM-1 have previously shown potentiated platelet aggregation and secretion responses to collagen and the GPVI-specific agonist collagen-related peptide (CRP) despite no alteration in platelet count [[10], [11], [12], [13], [14]]. This hyperactivity is also seen *in vivo*, with enhanced thrombus formation observed in response to laser and ferric chloride (FeCl_3_) injury models [15].

Recently, we have shown that the ITIM-containing high-affinity collagen receptor, leukocyte-associated immunoglobulin-like receptor-1 (LAIR-1), also inhibits collagen-mediated platelet and megakaryocyte (MK) activation and thrombus formation *in vivo* [16]. The inhibitory function of LAIR-1 has been demonstrated to also be mediated through association of Shp2, as well as Shp1 and Csk, although LAIR-1 is not expressed in platelets or mature MKs [[17], [18], [19]].

The aim of this study was to investigate the role of LAIR-1 alongside PECAM-1 in MK development and platelet production and determine any functional redundancy through the characterization of a LAIR-1/PECAM-1 double knockout (DKO) mouse model. We show that DKO mice exhibit thrombocytosis similarly to LAIR-1 knockout (KO) mice (25% increases in platelet counts, respectively, compared with wild-type [WT]). Furthermore, platelets from LAIR-1 KO, PECAM-1 KO, and DKO mice were equally hyperresponsive to collagen and exhibited increased α_IIb_β_3_ outside-in signaling, all of which are SFK-dependent and previously shown to be regulated by LAIR-1 and PECAM-1 [4,16,20]. Platelet surface expression of the collagen receptor GPVI and the integrins α_2_β_1_ and α_IIb_β_3_ were normal in all mouse models, suggesting that LAIR-1 and PECAM-1 regulate the same inhibitory pathway and potential alternative compensatory mechanisms, including the ITIM-containing coinhibitory receptor G6b-B [21,22]. We and others have previously shown that LAIR-1 and PECAM-1 KO mouse models exhibit an increase in thrombus formation *in vivo* in the FeCl_3_ injury model [15,16]. Interestingly, thrombus formation and platelet SFK activity were normal in DKO mice but previously reported to be enhanced in LAIR-1 KO mice [16]. This may be due to compensatory mechanisms that are only activated in the absence of LAIR-1 and PECAM-1 and deletion of both receptors in other hematopoietic and vascular lineages that contribute to thrombosis.

## 2 Methods

### 2.1 Animals

LAIR-1/PECAM-1 double-deficient mice were generated by mating LAIR-1- and PECAM-1-deficient mice, which were generated as previously stated [16,23]. All mice were on a C57BL/6 background. All procedures were in accordance with the Animals (Scientific Procedures) Act of 1986 and undertaken with United Kingdom Home Office approval.

### 2.2 Antibodies and reagents

All reagents were sourced from Sigma-Aldrich or as previously described [16].

### 2.3 Platelet aggregation, MK preparation, and proplatelet formation

Blood was collected from terminally CO_2_-narcosed mice from the abdominal vena cava into 1:10 (volume per volume) acid-citrate-dextrose anticoagulant. Washed platelets were prepared as previously described [16]. Platelet aggregation and adenosine triphosphate (ATP) secretion were simultaneously measured using a lumi-aggregometer (Chrono-Log). MK isolation, culture, and proplatelet formation assay were performed as previously described [16,24]. Proplatelet formation was quantified using a semiautomated machine learning–based Konstanz Information Miner workflow as previously described [[25], [26], [27]].

### 2.4 Flow cytometry

Surface protein expression was measured in whole blood or bone marrow-derived MKs with indicated fluorescein isothiocyanate-conjugated antibodies by flow cytometry (BD Accuri C6 for platelets; BD FACSCalibur for bone marrow cells), as previously described [16].

### 2.5 *In vivo* thrombosis assay

FeCl_3_ injury of the carotid artery was performed as previously described [16,28].

### 2.6 Biochemistry

Whole-cell lysates were either boiled in sodium dodecyl sulfate loading buffer and analyzed by sodium dodecyl sulfate–polyacrylamide gel electrophoresis and traditional Western blotting or analyzed on an automated capillary-based immunoassay platform Wes (ProteinSimple) for quantitative analysis as previously described [16,28].

### 2.7 Statistical analysis

Data are presented as mean ± SEM unless stated otherwise. Statistical significance was analyzed using analysis of variance, unpaired Student’s *t*-test, or the Mann–Whitney U-test. A *P* < .05 was considered statistically significant.

## 3 Results and Discussion

### 3.1 LAIR-1/PECAM-1 DKO mice have mild thrombocytosis and normal MK development

**Figure 1:**
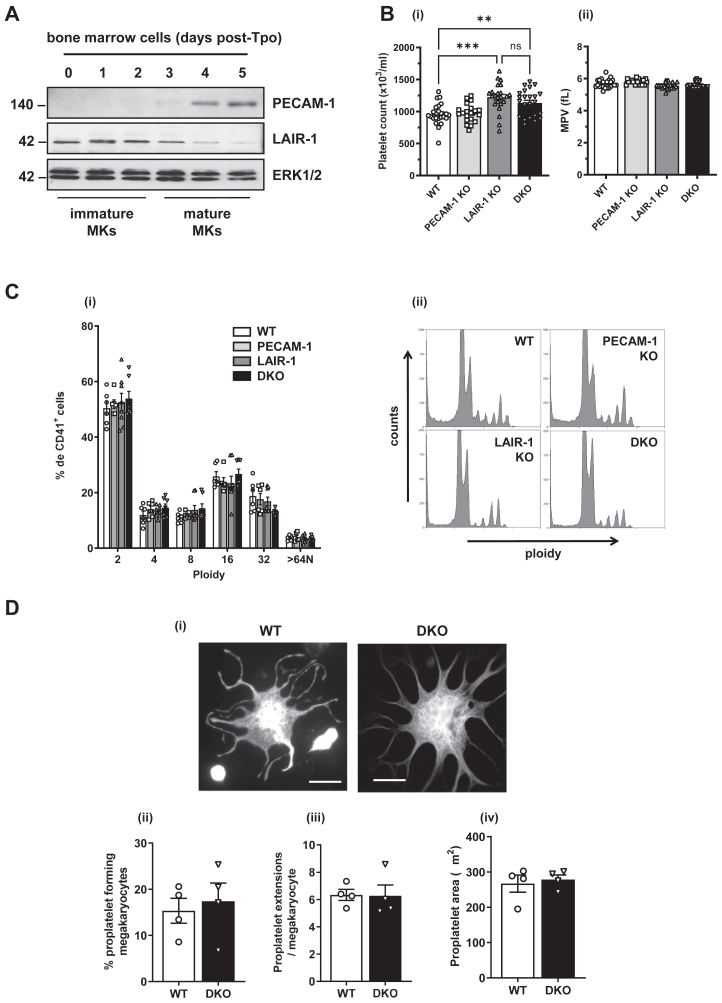
LAIR-1/PECAM-1 double knockout (DKO) mice exhibit a mild increase in platelet count. (A) LAIR-1 and PECAM-1 are inversely expressed during megakaryopoiesis. Whole-cell lysates were prepared of bone marrow cells from wild-type (WT) mice 0 to 5 days post culture in the presence of thrombopoietin (Tpo) and Western blotted for LAIR-1, PECAM-1, and ERK1/2. Representative blots from *n* = 3 independent experiments. (B) LAIR-1/PECAM-1 DKO mice exhibit mild thrombocytosis. (i) Platelet counts and (ii) volumes from PECAM-1 knockout (KO), LAIR-1 KO, LAIR-1/PECAM-1 DKO, and litter-matched WT mice were measured (*n* = 18-19 mice/genotype); mean ± SEM. (C) LAIR-1/PECAM-1 DKO mouse megakaryocytes (MKs) exhibit normal differentiation. The ploidy of mature bone marrow-derived MKs was quantified after propidium iodide staining using flow cytometry. (i) The percentage of 2-128N ploidy cells was quantified, and (ii) representative profiles are shown. Mean ± SEM; *n* = 6 to 8 mice/genotype. (D) LAIR-1/PECAM-1 DKO mouse MKs exhibit normal proplatelet formation. Mature bone marrow-derived MKs were plated on fibrinogen-coated surfaces (100 μg/mL, 5 hours, 37 °C, 5% CO_2_). (i) Representative images of tubulin-stained proplatelet-forming MKs were taken. Scale bar: 50 μm. (ii) Percent of MK forming proplatelets, (iii) number of extensions per proplatelet forming MK, and (iv) proplatelet area. Mean ± SEM; *n* = 200 to 250 MKs from 3 mice/genotype. MPV, mean platelet volume; ns, not significant. ∗∗*P* < .01; ∗∗∗*P* < .001.

We first analyzed the protein expression of LAIR-1 alongside PECAM-1 during mouse MK differentiation by Western blot. We show that LAIR-1 and PECAM-1 are inversely expressed during megakaryopoiesis. Indeed, LAIR-1 was expressed in immature MKs and decreased during maturation, whereas PECAM-1 expression increased as MKs matured (Figure 1A).

To investigate functional redundancy between LAIR-1 and PECAM-1, we generated LAIR-1/PECAM-1 DKO mice. Hematological analysis revealed that LAIR-1/PECAM-1 DKO mice displayed a mild thrombocytosis (25% increase in platelet count; Figure 1Bi), with unchanged platelet volume (Figure 1Bii) and platelet clearance (Supplementary Figure S1). We next investigated MK differentiation. We show that the absence of LAIR-1 and PECAM-1 had no effect on MK development as normal ploidy pattern was observed (Figure 1Ci, ii), demonstrating that neither LAIR-1 nor PECAM-1 regulates MK differentiation. To investigate the cause of thrombocytosis, we measured proplatelet formation *in vitro*. Surprisingly, MKs from DKO mice exhibited normal proplatelet formation, with number of MKs forming proplatelets and proplatelet surface area being unchanged (Figure 1Di–iv), which likely explains why the thrombocythemia in these mice is not as pronounced as in the LAIR-1 KO mice (20% vs 25% increases in DKO and LAIR-1 KO mice, respectively). In addition, surface receptor levels were also normal in DKO MKs (Supplementary Table S1).

### 3.2 LAIR-1/PECAM-1 DKO mice exhibit increased platelet reactivity *in vitro*

**Figure 2:**
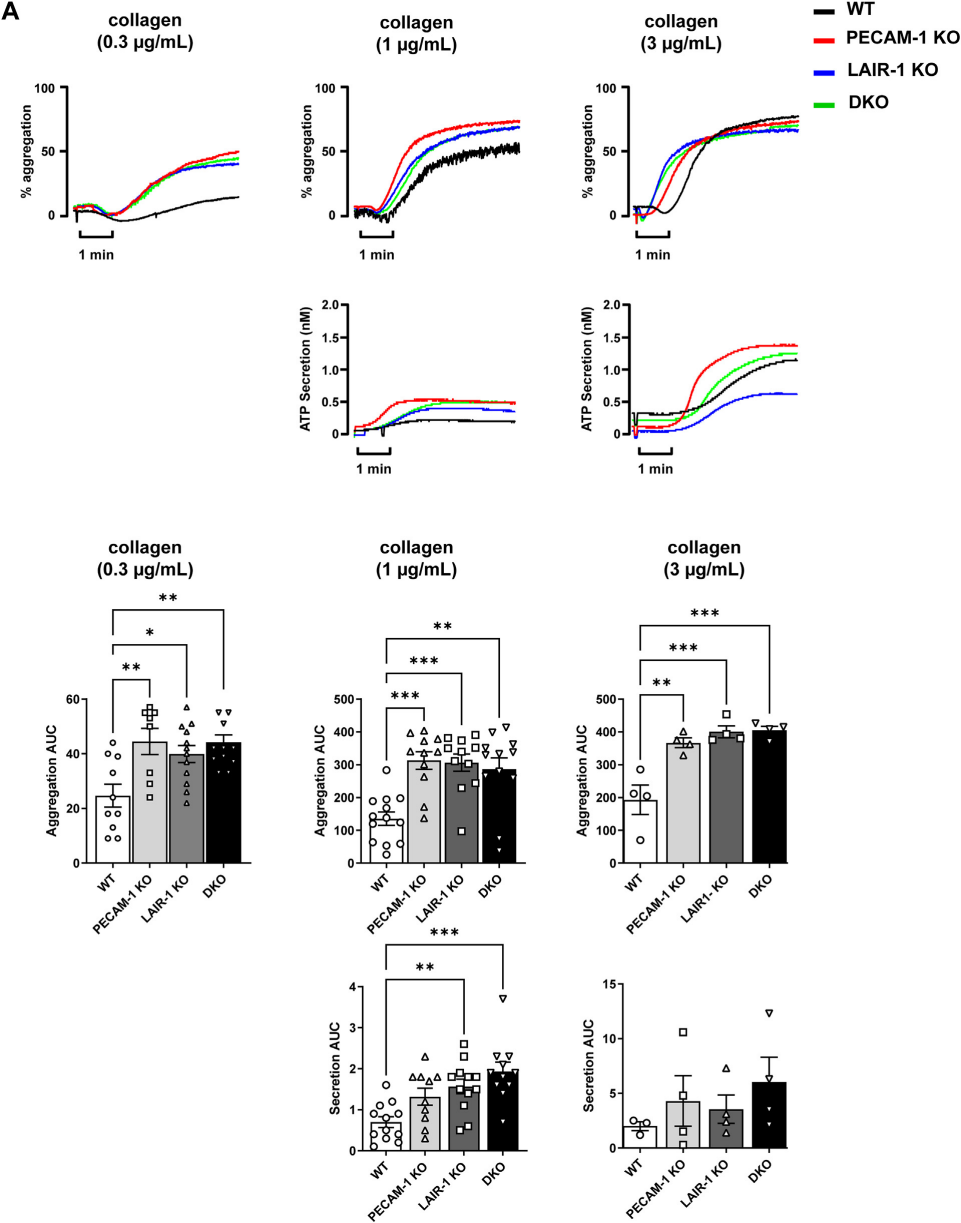

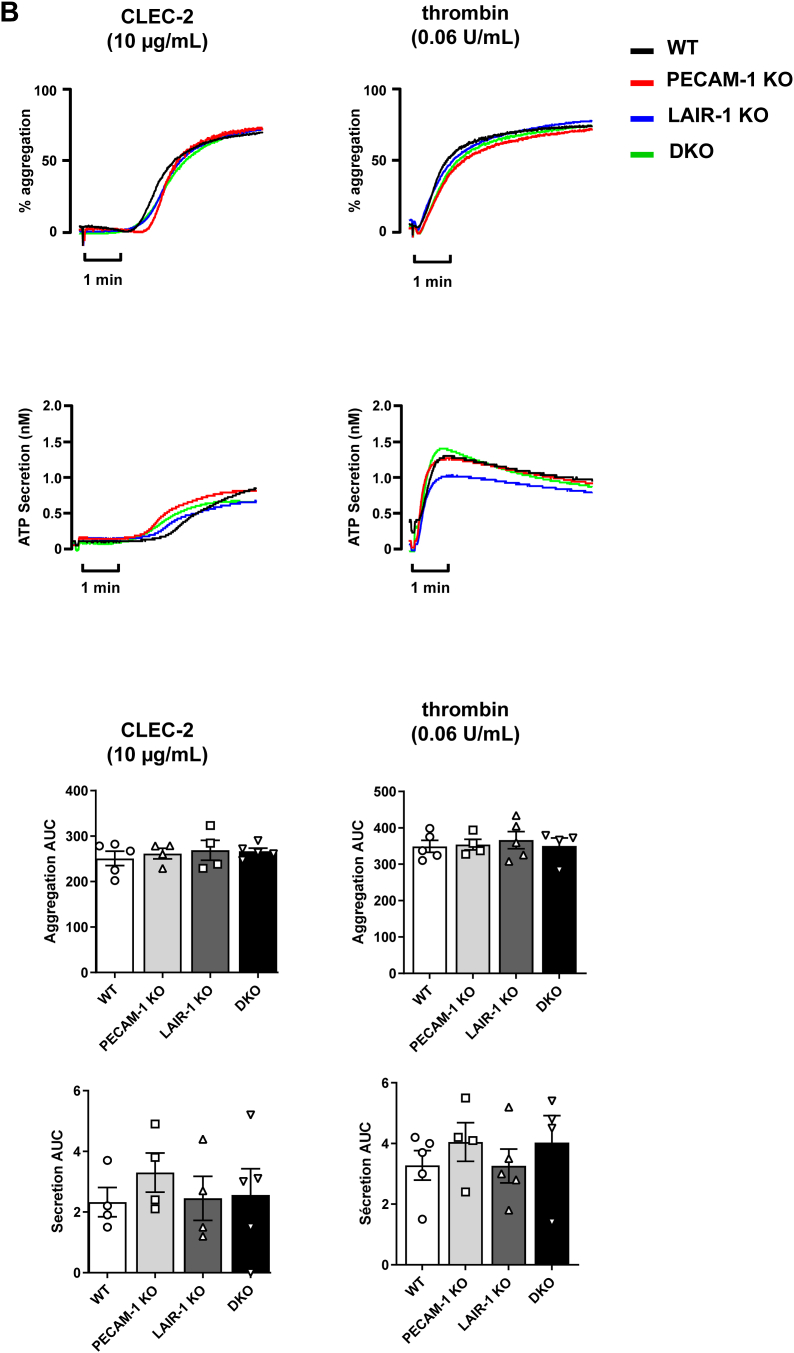
LAIR-1/PECAM-1 double knockout (DKO) mouse platelets exhibit increased reactivity *in vitro*. (A, B) Increased collagen-induced platelet aggregation. Washed platelets from PECAM-1 knockout (KO), LAIR-1 KO, LAIR-1/PECAM-1 DKO, and litter-matched wild-type (WT) mice were stimulated with 0.3, 1, or 3 μg/mL of (A) collagen, (B) collagen-related peptide, (C, D) 10 μg/mL CLEC-2 antibody, and 0.06 U/mL thrombin. The percentage of platelet aggregation and adenosine triphosphate (ATP) secretion (nanoMolar) were measured using a lumi-aggregometer. Average traces and area under the curve (AUC) quantification from 4 to 17 mice/genotype are represented *∗P* < .05; *∗∗P* < .01; *∗∗∗P* < .001.

To determine whether there is any functional redundancy between LAIR-1 and PECAM-1 in platelets, aggregation and ATP secretion were monitored in response to various agonists. Both LAIR-1 and PECAM-1 single KO mice have previously been demonstrated to have enhanced platelet aggregation to low-dose GPVI agonists, collagen, and CRP, which are abolished at higher concentrations [10,12,16]. Here, we show that aggregation responses to low-dose collagen (Figure 2A) and CRP (Supplementary Figure S2) were enhanced in platelets from DKO mice despite normal expression of collagen receptors GPVI and α_2_β_1_ (Supplementary Table S2). As expected, platelet aggregation in response to thrombin was unchanged (Figure 2B) as LAIR-1 and PECAM-1 modulate SFK-mediated GPVI activation but not PAR4 G-protein coupled receptor activation [16,22]. Interestingly, aggregation responses to CLEC-2, which is a hemi–ITAM-containing receptor and has a different triggering mechanism from that of GPVI-FcRγ [29], were normal. ATP secretion in response to agonists was also normal (Figure 2B).

Findings in this study reveal that absence of PECAM-1 alongside LAIR-1 does not result in further enhancement of platelet reactivity. DKO mouse platelet aggregation responses to GPVI agonists, collagen, and CRP were comparable with LAIR-1 and PECAM-1 single KO platelets. The absence of further enhancement of platelet reactivity suggests that the ITIM-containing receptors LAIR-1 and PECAM-1 participate in the same inhibitory pathway to reduce GPVI-mediated platelet activation. The lack of further enhancing effects in the absence of both receptors is potentially explained by the altered signaling in LAIR-1 KO mouse platelets, negating the inhibitory effect of PECAM-1.

**Figure 3:**
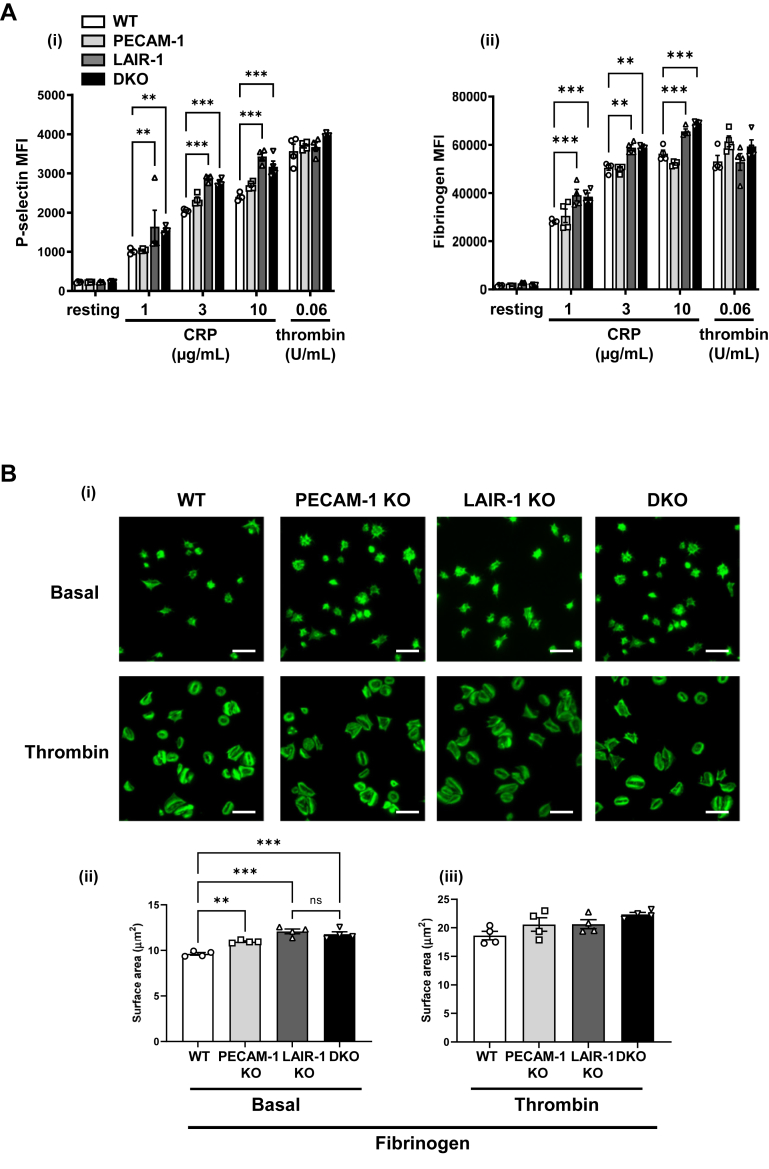

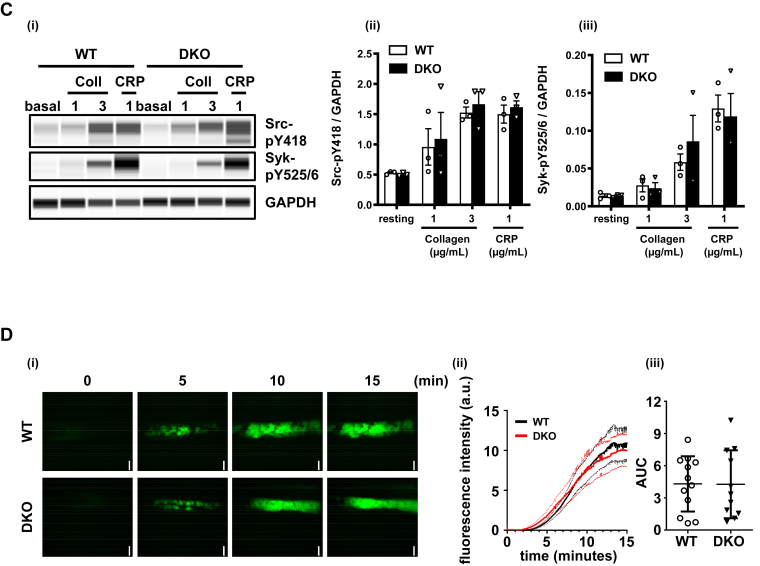
LAIR-1/PECAM-1 double knockout (DKO) mouse platelets exhibit increased P-selectin, platelet spreading on fibrinogen, and normal ferric chloride-induced thrombus formation *in vivo.* (A) Increased P-selectin surface expression and fibrinogen binding in LAIR-1/PECAM-1 DKO mouse platelets. (i) P-selectin surface expression and (ii) fibrinogen binding of platelets in response to 1, 3, and 10 μg/mL collagen-related peptide (CRP) and 0.06 U/mL thrombin were measured. Median fluorescence intensity (MFI) ± SEM is represented; *n* = 5 mice/genotype. (B) Increased platelet spreading in LAIR-1/PECAM-1 DKO mice. Basal and thrombin (0.1 U/mL)-activated platelets were plated on a fibrinogen-coated surface. (i) Representative phalloidin-stained images of platelets. (ii) Surface area of individual platelets was measured; *n* = 3 to 5 mice/genotype, 250 to 500 platelets/condition; mean ± SEM; scale bar: 5 μm. (C) Normal GPVI-mediated signaling in LAIR-1/PECAM-1 DKO platelets. Whole-cell lysates of basal and stimulated platelets (collagen [Coll] 1 and 3 μg/mL and CRP 1 μg/mL) were analyzed by capillary-based immunoassays with indicated antibodies. (i) Representative data displayed as blots and (i, ii) quantification of normalized peak area is shown as mean ± SEM; *n* = 3 independent experiments/genotype. (D) Normal ferric chloride-induced thrombus formation *in vivo* in LAIR-1/PECAM-1 DKO mice. Mice were injected with DyLight488-conjugated anti-GPIbβ antibody (0.1 μg/g body weight, X488). Exposed carotid arteries were injured with 10% ferric chloride for 3 minutes, and the accumulation of platelets (green) into the thrombi was assessed. (i) Representative fluorescence images from X488-labeled platelets after ferric chloride injury of the carotid artery are shown. Scale bar: 200 μm. (ii) Each curve represents the mean integrated fluorescence density ± SEM in arbitrary units (a.u.) for 11 to 12 mice/genotype. (iii) The area under the curve (AUC) of the integrated fluorescence density is represented (mean ± SEM). See also Supplementary Videos S1 and S2. KO, knockout; WT wild-type. ∗∗*P* < .01; ∗∗∗*P* < .001.

To determine whether LAIR-1 and PECAM-1 affect α-granule secretion and integrin activation, P-selectin expression and fibrinogen binding were assessed in the various KO mouse models. Consistent with previously published findings, PECAM-1–deficient platelets displayed normal P-selectin exposure and fibrinogen binding, and platelets from LAIR-1 KO mice exhibited increased α-granule secretion and integrin activation [12,16]. In agreement with aggregation findings, P-selectin exposure and fibrinogen binding were increased in platelets from DKO mice in response to CRP (Figure 3Ai, ii).

We next investigated platelet spreading on a fibrinogen-coated surface as this is an important aspect of platelet function that is dependent on the integrin α_IIb_β_3_ for adhesion and cytoskeletal remodeling [30]. Platelets from PECAM-1 KO and LAIR-1 KO mice exhibited an increase in spreading on fibrinogen in comparison with WT platelets (Figure 3Bi, ii), as previously shown [10,16]. Interestingly, platelets from DKO mice exhibited a comparable increase in spreading with LAIR-1 and PECAM-1 single KO platelets. Furthermore, when preactivated with 0.1 U/mL thrombin, platelets from all mice spread normally (Figure 3Bi, iii). The absence of further enhancing effects in DKO mice potentially suggests that both receptors participate in the same inhibitory pathway.

We have previously shown that increased SFK activity was the underlying cause of enhanced thrombosis in LAIR-1–deficient mice [16]. We therefore assessed phosphorylation of tyrosine residue 418 in the activation loop of Src (Src p-Tyr418) and tyrosine residues 525/526 in the activation loop of Syk (Syk p-Tyr525/526) as indirect markers of activation before and after collagen and CRP stimulation of platelets. Intriguingly, we found that DKO platelets exhibited normal SFK and Syk tyrosine phosphorylation in resting and activated platelets (Figure 3Ci–iii), correlating with the normal thrombosis response in DKO mice. In platelets from LAIR-1 KO mice, the increased activation of SFKs results in increased phosphorylation of ITAMs and their downstream signaling molecules, which would overcome the modest inhibitory effect of PECAM-1, as is the case at higher agonist concentrations.

Further investigation is however necessary to explain the loss of enhanced SFK activity in DKO mice. LAIR-1 and PECAM-1 have been shown to interact with similar signaling proteins to mediate their inhibitory effects. One explanation for the loss of enhanced SFK activation might be altered expression of the LAIR-1 and PECAM-1 binding partner and regulator of SFK activity Csk or its homolog Chk [9,18]. However, this was not the case in LAIR-1/PECAM-1 DKO platelets and MKs, which contained comparable levels of Csk and Chk as control platelets (Supplementary Figure S3). Other potential candidates include the protein tyrosine phosphatases CD148, Shp1, Shp2, and protein tyrosine phosphatases-1B, all of which have been implicated in regulating SFK activity [28,31].

We next investigated the physiological effects of ablating both LAIR-1 and PECAM-1 on tail bleeding and thrombus formation *in vivo*, utilizing the FeCl_3_-injury model, with which both LAIR-1 and PECAM-1 single KO mice have been shown to have increased thrombosis [15,16]. DKO mice did not exhibit a bleeding diathesis after tail injury (Supplementary Figure S4). Following application of 10% FeCl_3_ for 3 minutes to the carotid artery, surprisingly, DKO mice formed thrombi comparable with control mice (Figure 3Di–iii). Although enhanced thrombosis to FeCl_3_ injury was also previously reported in PECAM-1 KO mice, the authors concluded the increase was modest, as several WT mice demonstrated equivalent thrombosis [15]. In addition, in the study by Falati et al. [15], thrombosis was assessed using a Doppler flow probe rather than fluorescence intensity, as utilized in this and the LAIR-1 KO study, which may contribute to reported differences.

In conclusion, analysis of platelets and MKs from KO and DKO mouse models in parallel revealed that LAIR-1/PECAM-1 DKO mice have mild thrombocytosis and produce platelets that are hyperresponsive to GPVI-mediated platelet activation. These phenotypes were comparable with those observed in single KO platelets, demonstrating that LAIR-1 and PECAM-1 participate in the same inhibitory pathway and do not have additive or synergistic functions. Interestingly, thrombus formation and platelet SFK activity appear to be rescued in DKO mice, whereas previously reported to be enhanced in LAIR-1 KO mice [16]. This may be due to compensatory mechanisms that are only activated in the absence of LAIR-1 and PECAM-1. Furthermore, deletion of both receptors in other hematopoietic and vascular lineages that contribute to thrombosis may also explain the different phenotypes between the single- and double-deficient mouse models.
