# Supplementary material for: LAIR-1 and PECAM-1 function via the same signaling pathway to inhibit GPVI-mediated platelet activation
Source: Res Pract Thromb Haemost. 2024 Aug 23;8(6):102557. doi: 10.1016/j.rpth.2024.102557 (PMC11421324; doi:10.1016/j.rpth.2024.102557)
Supplement: Supplementary Material [file mmc1.pptx]

## Slide 1
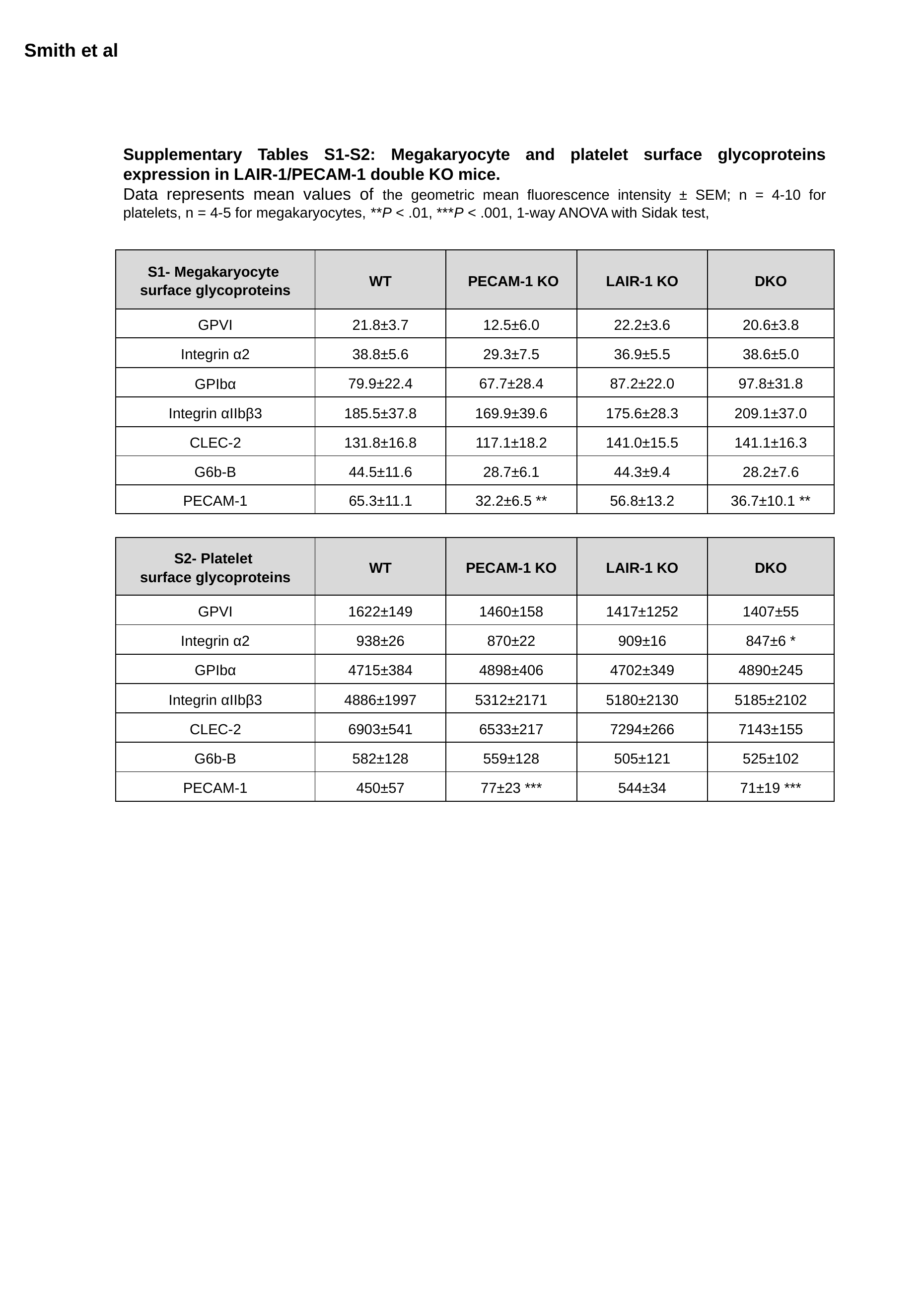

Smith et al
Supplementary Tables S1-S2: Megakaryocyte and platelet surface glycoproteins expression in LAIR-1/PECAM-1 double KO mice.
Data represents mean values of the geometric mean fluorescence intensity ± SEM; n = 4-10 for platelets, n = 4-5 for megakaryocytes, **P < .01, ***P < .001, 1-way ANOVA with Sidak test,
| S1- Megakaryocyte surface glycoproteins | WT | PECAM-1 KO | LAIR-1 KO | DKO |
| --- | --- | --- | --- | --- |
| GPVI | 21.8±3.7 | 12.5±6.0 | 22.2±3.6 | 20.6±3.8 |
| Integrin α2 | 38.8±5.6 | 29.3±7.5 | 36.9±5.5 | 38.6±5.0 |
| GPIbα | 79.9±22.4 | 67.7±28.4 | 87.2±22.0 | 97.8±31.8 |
| Integrin αIIbβ3 | 185.5±37.8 | 169.9±39.6 | 175.6±28.3 | 209.1±37.0 |
| CLEC-2 | 131.8±16.8 | 117.1±18.2 | 141.0±15.5 | 141.1±16.3 |
| G6b-B | 44.5±11.6 | 28.7±6.1 | 44.3±9.4 | 28.2±7.6 |
| PECAM-1 | 65.3±11.1 | 32.2±6.5 \*\* | 56.8±13.2 | 36.7±10.1 \*\* |
| S2- Platelet surface glycoproteins | WT | PECAM-1 KO | LAIR-1 KO | DKO |
| --- | --- | --- | --- | --- |
| GPVI | 1622±149 | 1460±158 | 1417±1252 | 1407±55 |
| Integrin α2 | 938±26 | 870±22 | 909±16 | 847±6 \* |
| GPIbα | 4715±384 | 4898±406 | 4702±349 | 4890±245 |
| Integrin αIIbβ3 | 4886±1997 | 5312±2171 | 5180±2130 | 5185±2102 |
| CLEC-2 | 6903±541 | 6533±217 | 7294±266 | 7143±155 |
| G6b-B | 582±128 | 559±128 | 505±121 | 525±102 |
| PECAM-1 | 450±57 | 77±23 \*\*\* | 544±34 | 71±19 \*\*\* |

## Slide 2
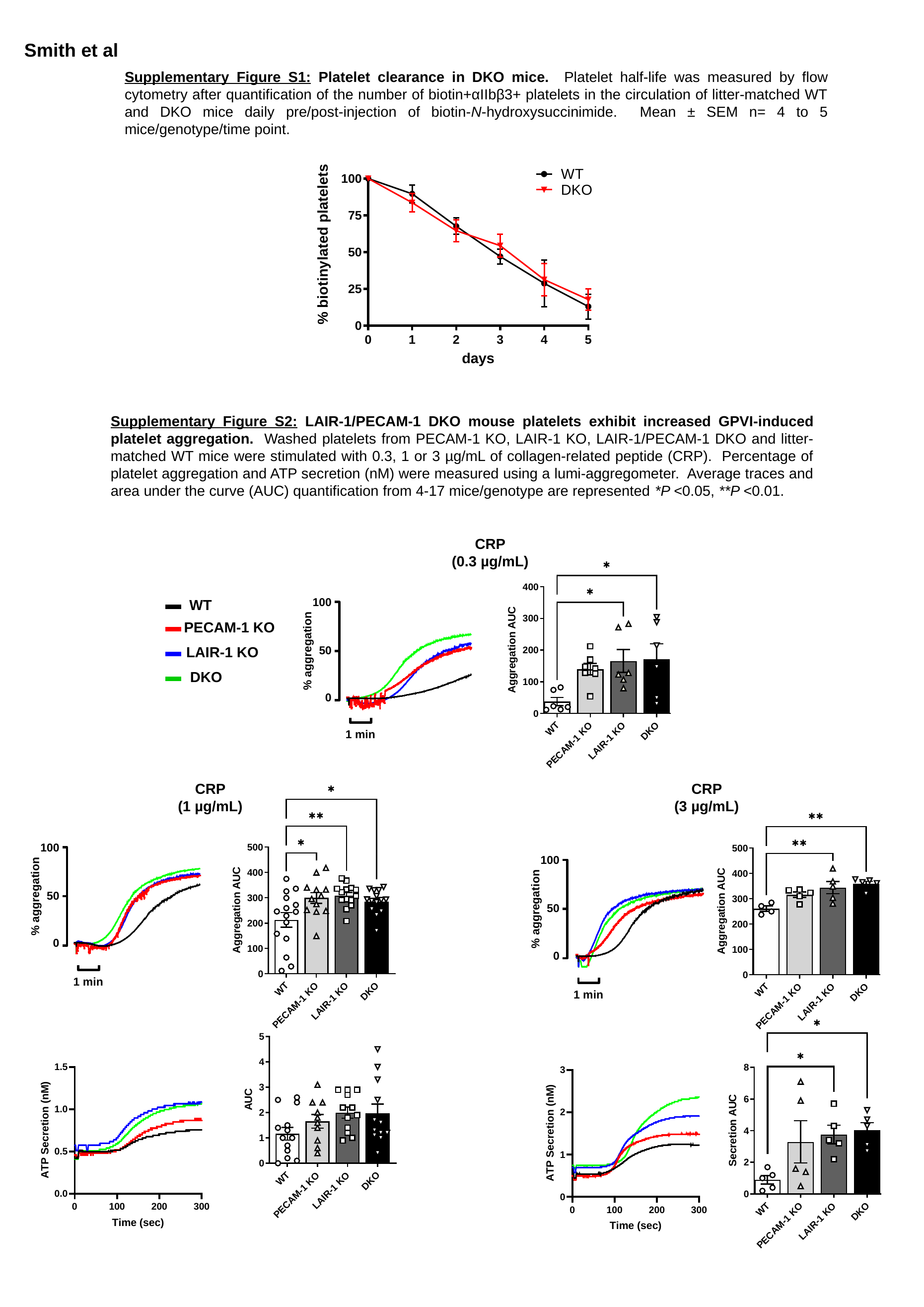

Smith et al
Supplementary Figure S1: Platelet clearance in DKO mice. Platelet half-life was measured by flow cytometry after quantification of the number of biotin+αIIbβ3+ platelets in the circulation of litter-matched WT and DKO mice daily pre/post-injection of biotin-N-hydroxysuccinimide. Mean ± SEM n= 4 to 5 mice/genotype/time point.
Supplementary Figure S2: LAIR-1/PECAM-1 DKO mouse platelets exhibit increased GPVI-induced platelet aggregation. Washed platelets from PECAM-1 KO, LAIR-1 KO, LAIR-1/PECAM-1 DKO and litter-matched WT mice were stimulated with 0.3, 1 or 3 µg/mL of collagen-related peptide (CRP). Percentage of platelet aggregation and ATP secretion (nM) were measured using a lumi-aggregometer. Average traces and area under the curve (AUC) quantification from 4-17 mice/genotype are represented *P <0.05, **P <0.01.
CRP
(0.3 µg/mL)
WT
PECAM-1 KO
LAIR-1 KO
DKO
0
50
100
% aggregation
1 min
CRP
(1 µg/mL)
CRP
(3 µg/mL)
0
50
100
% aggregation
1 min
0
50
100
% aggregation
1 min

## Slide 3
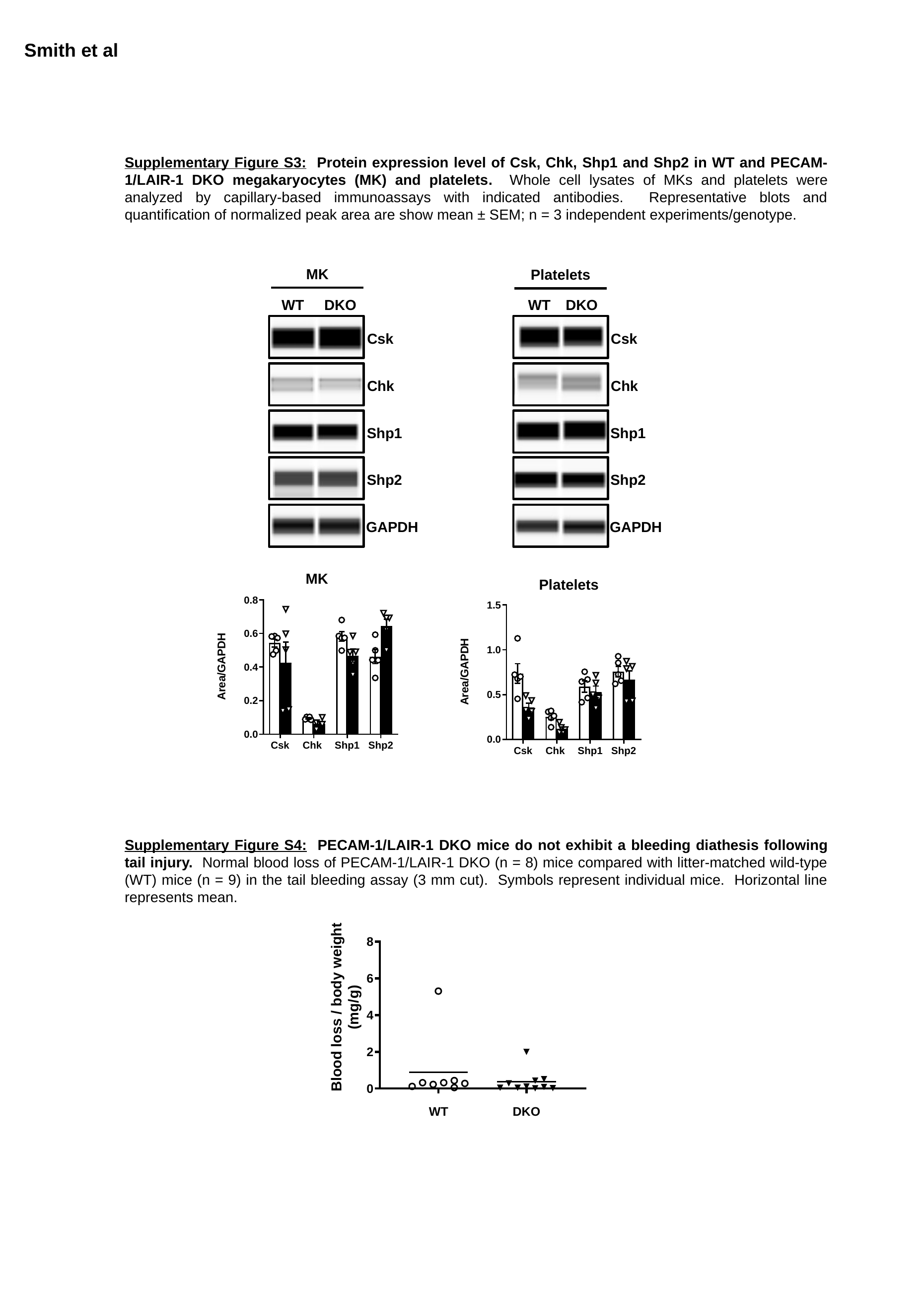

Smith et al
Supplementary Figure S3: Protein expression level of Csk, Chk, Shp1 and Shp2 in WT and PECAM-1/LAIR-1 DKO megakaryocytes (MK) and platelets. Whole cell lysates of MKs and platelets were analyzed by capillary-based immunoassays with indicated antibodies. Representative blots and quantification of normalized peak area are show mean ± SEM; n = 3 independent experiments/genotype.
MK
Platelets
WT
DKO
WT
DKO
Csk
Csk
Chk
Chk
Shp1
Shp1
Shp2
Shp2
GAPDH
GAPDH
MK
Platelets
Supplementary Figure S4: PECAM-1/LAIR-1 DKO mice do not exhibit a bleeding diathesis following tail injury. Normal blood loss of PECAM-1/LAIR-1 DKO (n = 8) mice compared with litter-matched wild-type (WT) mice (n = 9) in the tail bleeding assay (3 mm cut). Symbols represent individual mice. Horizontal line represents mean.
